# Successful Anesthetic Management Using Remimazolam in a Patient With Spinocerebellar Degeneration Undergoing Pulmonary Resection

**DOI:** 10.7759/cureus.100170

**Published:** 2025-12-27

**Authors:** Yasuhiro Watanabe, Takamitsu Hayakawa, Hirofumi Okabe

**Affiliations:** 1 Department of Anesthesia, Japanese Red Cross Shizuoka Hospital, Shizuoka, JPN; 2 Department of Thoracic Surgery, Japanese Red Cross Shizuoka Hospital, Shizuoka, JPN

**Keywords:** aspiration, cerebellar ataxia, cortical cerebellar atrophy, dysphagia, pulmonary resection, remimazolam, spinocerebellar degeneration, supraglottic airway

## Abstract

Spinocerebellar degeneration (SCD) comprises a broad range of neurodegenerative disorders mainly characterized by cerebellar ataxia, and the prevention of perioperative respiratory complications is of great importance in patients with cerebellar ataxia. Clinical papers addressing anesthetic management using remimazolam in patients with SCD, specifically those undergoing pulmonary resection, are limited. A 79-year-old man with cortical cerebellar atrophy, a sporadic adult-onset SCD, was admitted for pulmonary resection for primary lung adenocarcinoma. He has experienced chronic aspiration caused by dysphagia, placing him at an increased risk for perioperative respiratory complications. Video-assisted right S6 segmentectomy was performed under general-epidural anesthesia using remimazolam, remifentanil, and local anesthetics. During emergence from anesthesia and tracheal extubation, a supraglottic airway was inserted while the double-lumen tube remained in place, after which the tube was removed under deep anesthesia. Subsequently, continuous infusion of remimazolam and remifentanil was terminated, and remimazolam was antagonized using flumazenil. After confirmation of spontaneous breathing and response to verbal commands, the supraglottic airway was removed without triggering the cough reflex or laryngospasm. The patient was fully conscious and did not experience re-sedation or respiratory depression. Furthermore, he had a good postoperative course and was discharged without respiratory complications or exacerbation of neurological symptoms. In conclusion, anesthetic management using remimazolam alongside careful airway management contributed to the successful management of a patient with SCD undergoing pulmonary resection. For a patient exhibiting cerebellar ataxia, remimazolam can be a useful anesthetic agent.

## Introduction

Spinocerebellar degeneration (SCD) refers to a broad range of neurodegenerative disorders mainly characterized by motor ataxia, which is diagnosed by excluding secondary causes, including malignancy, infection, vascular diseases, and autoimmune disorders [1]. Among them, cortical cerebellar atrophy (CCA) is a sporadic SCD subtype, neuropathologically characterized by the degeneration and death of Purkinje cells in the cerebellar cortex, presenting as an adult-onset, gradually progressive cerebellar ataxia [1]. In patients with cerebellar ataxia, particularly those suffering from dysphasia-related chronic aspiration, the prevention of perioperative respiratory complications is of great importance [2].

Remimazolam, an ultrashort-acting intravenous benzodiazepine that has been recently approved for clinical use, offers the fast onset and offset of sedation, as well as a predictable duration of action [3]. Despite reports of successful management using various anesthetic techniques in patients with SCD [2,4-7], there are no clinical papers addressing anesthetic management using remimazolam in such patients, specifically those undergoing pulmonary resection. We herein report successful remimazolam-based anesthetic management combined with careful airway management in a patient with CCA undergoing video-assisted thoracic surgery.

## Case presentation

**Figure 1:**
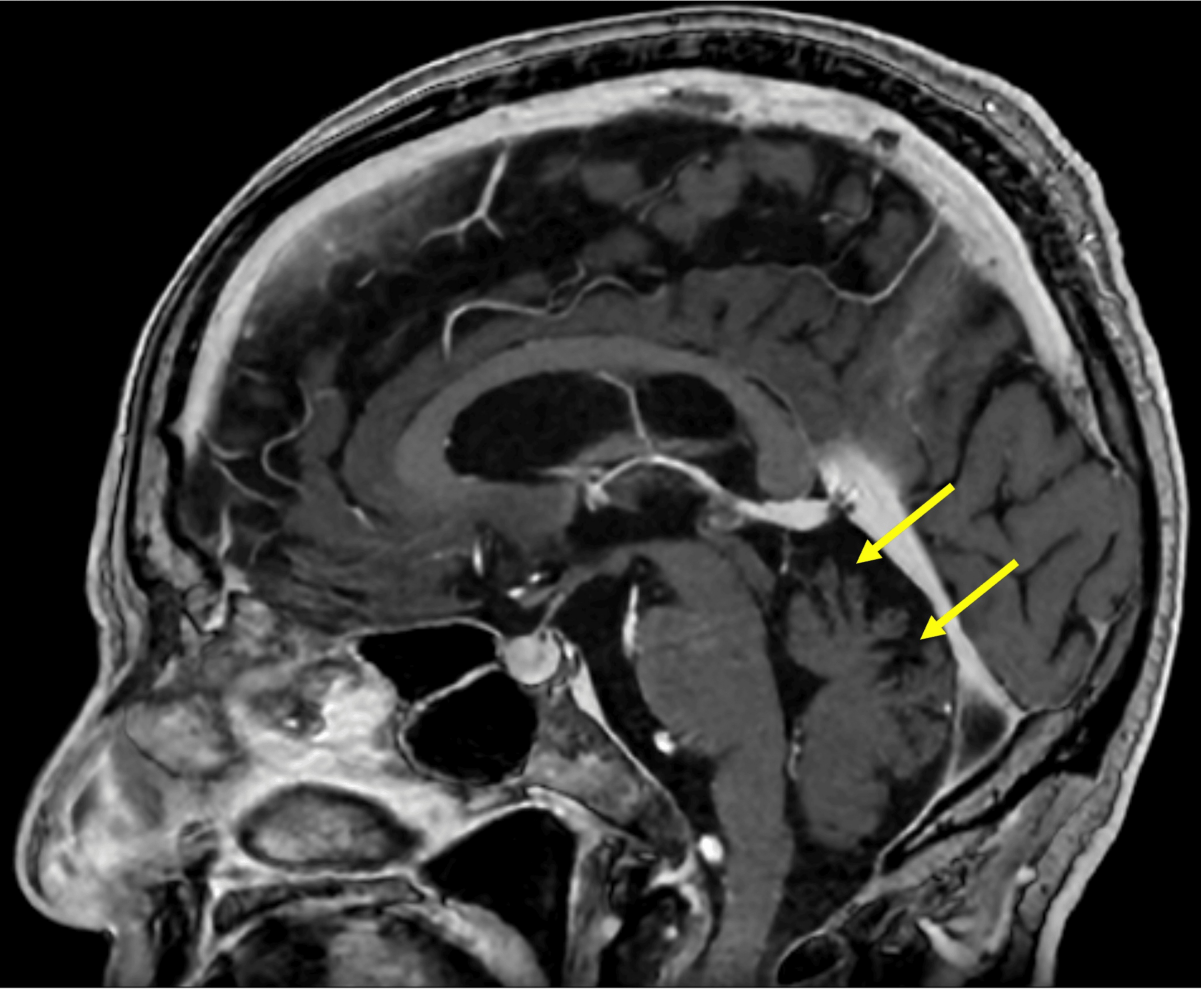
Magnetic resonance imaging of the head Gadolinium-enhanced magnetic resonance imaging that was performed one month prior to the surgery clearly demonstrates atrophy and foliation of the cerebellar cortex (arrows).

A 79-year-old man (height, 156.3 cm; weight, 45 kg; body mass index, 18.4) without a history of smoking was admitted with primary lung adenocarcinoma. He was diagnosed with CCA 15 years ago based on ataxic symptoms in the limbs and the trunk, dysarthria, and saccadic ocular movements that had lasted for four years, along with cerebellar atrophy on magnetic resonance imaging (Figure 1). 

**Figure 2:**
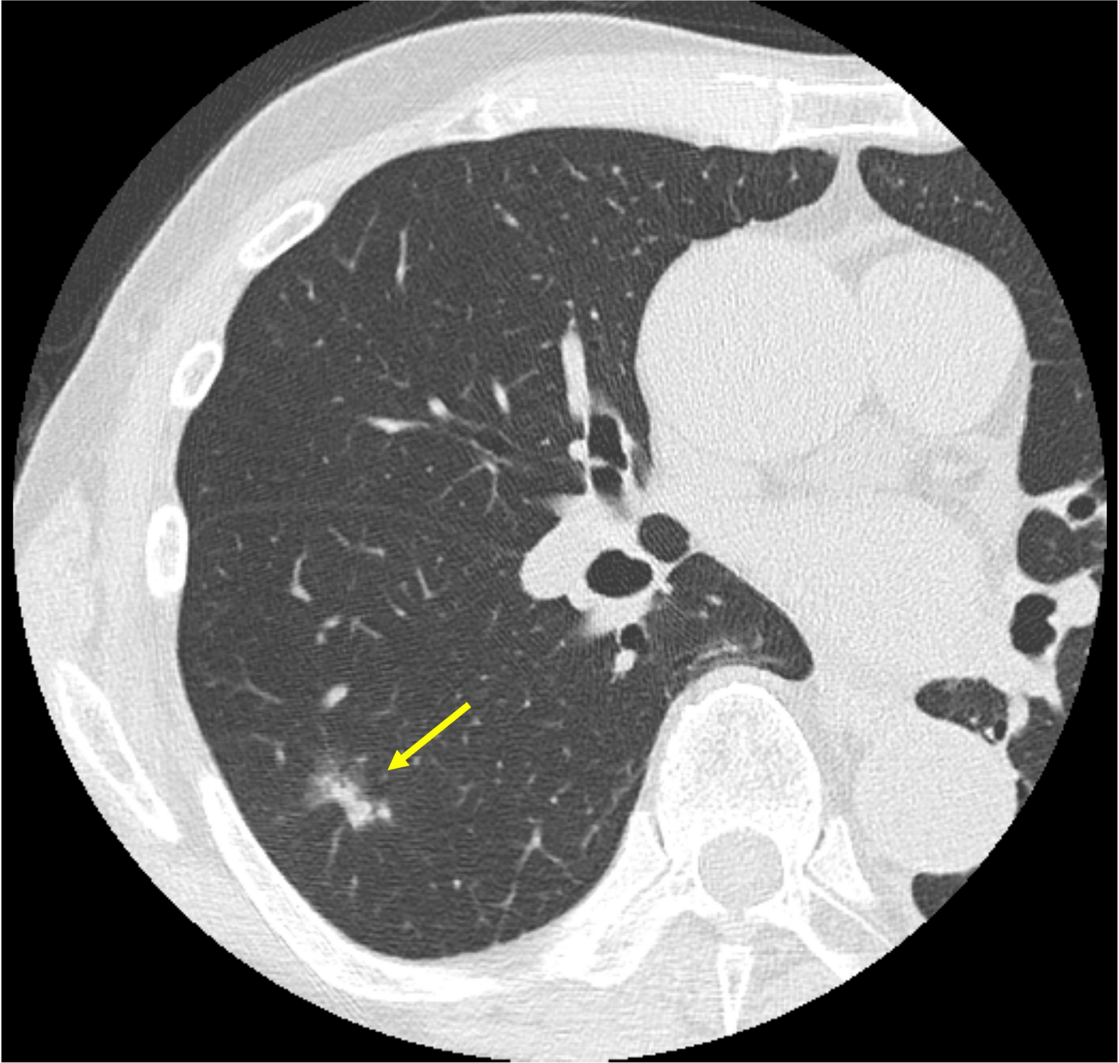
Chest computed tomography Chest computed tomography shows solitary lung adenocarcinoma in the right S6 segment (arrow). In contrast, there are no findings of interstitial lung disease.

As regards family history, he had 12 siblings, one of whom had dysarthria. Preoperative assessment revealed typical cerebellar symptoms, including swallowing difficulty, scanning speech, and ataxic gait. In addition, he has experienced dysphagia-related chronic aspiration, accompanied by a cough reflex. Nevertheless, dysautonomia, such as orthostatic hypotension and genitourinary impairment, as well as Parkinsonism, were not observed. Chest radiography showed a nodular shadow in the right upper quadrant, and chest computed tomography (CT) detected a 14.6 × 10 mm solitary nodule in the right S6 segment (Figure 2). 

Electrocardiography revealed sinus rhythm, whereas transthoracic echocardiography showed no wall motion abnormalities with a 72% ejection fraction. While the exact reason for the high ejection fraction was unknown, the patient did not have mitral regurgitation or diastolic dysfunction, as well as hyperthyroidism, severe anemia, or arteriovenous fistula. A pulmonary function test demonstrated a vital capacity of 2.31 L, corresponding to 79% of the predicted value. This restrictive ventilatory impairment was considered to be caused by CCA and kyphosis, as no comorbid conditions associated with restrictive lung disease, such as interstitial lung disease, were identified on CT imaging. The preoperative forced expiratory volume in 1 second percent predicted (FEV1.0) was 1.76 L, and the predicted postoperative FEV1.0 after right S6 segmentectomy was calculated to be 1.66 L, which was considered to be adequately preserved. The patient was not taking any oral medications, and the results of the blood test were unremarkable.

In the operating room, the patient’s blood pressure was 155/81 mmHg; heart rate, 57 beats per minute; and percutaneous oxygen saturation (SpO2), 96% in room air. In addition to standard monitoring (electrocardiography, non-invasive intermittent blood pressure measurement, SpO2, end-tidal carbon dioxide, and body temperature), anesthetic depth (BIS™; Medtronic, Minneapolis, MN, USA), neuromuscular monitoring at the adductor pollicis (TOF-Watch® SX; Organon, Swords, Dublin, Ireland), and invasive arterial blood pressure were monitored. An epidural catheter was inserted at the T6−T7 intervertebral space, and general anesthesia was induced using remifentanil 0.4 μg kg−1 min−1, remimazolam 7 mg (0.16 mg kg−1), followed by a continuous infusion of 1.0 mg kg−1 hr−1, and rocuronium bromide 40 mg. Following tracheal intubation with a 37-Fr left-sided double-lumen endobronchial tube, left-sided one-lung ventilation (OLV) was initiated using pressure-controlled ventilation with a peak pressure of 16 cmH₂O, positive end-expiratory pressure of 3 cmH₂O, respiratory rate of 14, and a fraction of inspired oxygen (FiO₂) of 1.0. Anesthesia was maintained using remimazolam 0.6−1.0 mg kg−1 hr−1, remifentanil 0.1−0.15 μg kg−1 min−1, intermittent administration of rocuronium bromide 10 mg to maintain the condition of neuromuscular blockade at a train-of-four count of 1, and intermittent epidural administration of 1% lidocaine (6 mL) and 0.25% levobupivacaine (10 mL) by keeping the BIS™ at 40−60. FiO2 was gradually decreased to 0.7, and intraoperative vital signs, including SpO2, were stabilized, requiring two bolus doses of ephedrine 4 mg. Fentanyl 50 μg was administered at 31 minutes and again at 3 minutes before the end of surgery as a transitional opioid for preventing rigidity and shivering. Ondansetron hydrochloride hydrate 4 mg was also administered during surgical closure to prevent postoperative nausea and vomiting (PONV). The infusion rate of remimazolam was decreased to 0.7 mg kg−1 hr−1 at 31 minutes and to 0.6 mg kg−1 hr−1 at 18 minutes before termination. After video-assisted right S6 segmentectomy with mediastinal nodal dissection was successfully performed, a supraglottic airway (i-gel™ #4; INTERSURGICAL®, Wokingham, Berkshire, UK) was inserted while the double-lumen tube (DLT) remained in place. Subsequently, the DLT was removed under deep anesthesia, and the infusion of remimazolam and remifentanil was terminated. Immediately after that, neuromuscular blockade (train-of-four count of 1) was reversed using sugammadex 140 mg. 

Four minutes after the discontinuation of remimazolam, flumazenil 0.5 mg was administered, and after another 8 minutes, with confirmation of spontaneous breathing with a tidal volume of 250−280 mL and response to verbal commands, i-gel™ was removed without triggering coughing or laryngospasm. The patient was fully conscious and did not experience postoperative delirium and PONV, as well as re-sedation. The durations of OLV and surgery were 235 and 239 minutes, respectively, and anesthesia lasted 309 minutes. The patients received 1,600 mL of crystalloid infusion. The blood loss was 30 mL, and the urine output was 250 mL. Postoperative pain control was achieved via patient-controlled epidural analgesia using 0.2% ropivacaine. Aside from an accidental removal of the thoracic drainage catheter on the next day of surgery, the postoperative course was uneventful, and the patient was discharged on postoperative day six without respiratory complications or exacerbation of neurological symptoms.

## Discussion

To our knowledge, this is the first clinical report addressing successful anesthetic management using remimazolam in a patient with SCD undergoing pulmonary resection. The present case highlighted the pivotal role of careful assessment of cerebellar ataxia and airway management to prevent perioperative respiratory complications.

In patients undergoing pulmonary resection, perioperative aspiration can result not only in pneumonia but also in cough reflex and resultant pneumothorax that compromise the postoperative course. In the present case of chronic aspiration, avoiding residual anesthetic agents was crucial. In view of maintaining oxygenation during OLV, the use of volatile anesthetics, which inhibit hypoxic pulmonary vasoconstriction in a dose-dependent manner, was excluded [8]. It is well recognized that some older patients undergoing thoracic surgery develop postoperative delirium [9]. A recent report indicated that the incidence of postoperative delirium did not differ between remimazolam and propofol used as sedative agents in general anesthesia [10]. Therefore, remimazolam was selected mainly for its property as an ultrashort-acting benzodiazepine that can be antagonized by flumazenil. It is recommended that anesthetic induction via continuous infusion of remimazolam at a dose of 6 or 12 mg kg−1 hr−1 be performed; however, it takes an average of 102 and 88.7 seconds, respectively, until the loss of consciousness [11]. In the present case, remimazolam was administered as a bolus injection to avoid the semi-awake state. Despite being disadvantageous in terms of muscle relaxation, thoracic epidural anesthesia was selected rather than continuous infusion of opioids for postoperative analgesia to avoid PONV, which would increase the risk of aspiration.

It is reported that the elimination half-life and terminal elimination half-life of remimazolam are between 7−11 and 37−53 minutes, respectively. In addition, the context-sensitive half-time of remimazolam is less than 10 minutes even after 4 hours of continuous infusion [12]. However, both an excessive dose of remimazolam and a high dose of flumazenil carry the risk of re-sedation 25−45 minutes after the flumazenil administration [13,14]. Herein, the dosage of remimazolam infusion was adjusted by referring to the BIS™ value. In contrast, the dosage and timing of flumazenil administration to antagonize remimazolam have not been fully established. We prioritized early removal of i-gel™, and in reality, the patient developed neither re-sedation nor respiratory depression after the administration of 0.5 mg flumazenil; however, for more reliable and robust postoperative management, it would have been better if we had administered an initial dose of 0.2 mg after confirming a certain degree of awareness [3,13].

Extubation of DLT has been associated with a higher incidence of respiratory complications [15]. To prevent lung injury during emergence from anesthesia and tracheal extubation, the DLT was replaced with i-gel™ in accordance with a method described by Arime et al. [16], where i-gel™ was inserted while the DLT was left in place. This enabled confirmation of the position of the inserted i-gel™ before the DLT removal. The fiberscope inserted through the ventilation port of i-gel™ observed the DLT being inserted between the vocal cords, indicating that i-gel™ was correctly positioned [16]. In fact, after the DLT removal, positive pressure ventilation via i-gel™ was easily achieved, not requiring an adjustment of the position. Another method, in which a supraglottic airway is inserted after DLT removal, may lead to failed ventilation and even re-intubation if the supraglottic airway is malpositioned, predisposing the patient to an elevated risk for respiratory complications.

The present report has several limitations. First, as regards the family history of the patient, one of his 12 siblings had dysarthria, and further genetic analysis had not been conducted. It was reported that genetic analysis of patients initially considered to have sporadic SCD revealed that 10−20% of them had hereditary SCD [17]. Therefore, hereditary SCD cannot be ruled out based solely on family history. Second, to date, evidence on the muscle relaxant effects of remimazolam and their antagonism by flumazenil remains limited. Although the use of remimazolam is not contraindicated in patients with neurodegenerative disorders, the validity should be carefully considered, taking into account their pathophysiology and clinical symptoms. 

## Conclusions

In patients with ataxic symptoms such as dysphasia-related aspiration, the prevention of perioperative respiratory complications is of great importance. General−epidural anesthesia using remimazolam alongside meticulous airway management contributed to the successful management of a patient with SCD undergoing pulmonary resection. Upon careful consideration, remimazolam can be a useful anesthetic agent in a patient with cerebellar ataxia.
